# Evolutionary history and functional diversification of *WOX* genes in *Annona Atemoya*

**DOI:** 10.1186/s12870-026-08109-5

**Published:** 2026-01-21

**Authors:** Xueyu Zhang, Xiaohong Dai, Minmin Jing, Shuailei Gu, Zhihui Chen, Jingjing Chen

**Affiliations:** 1 State Key Laboratory of Tropical Crop Breeding, South Subtropical Crops Research Institute, Chinese Academy of Tropical Agricultural Sciences, Sanya, Hainan 572024 China; 2 Key Laboratory of Tropical Fruit Biology, Ministry of Agriculture and Rural Affairs, South Subtropical Crops Research Institute, Chinese Academy of Tropical Agricultural Sciences, Zhanjiang, Guangdong 524091 China

**Keywords:** Annona, *WOX* gene family, Expression pattern, Phylogenetic analysis, Transcription factor

## Abstract

**Background:**

* WUSCHEL*-related homeobox (*WOX*) transcription factors play pivotal roles in plant development and evolutionary innovation. However, their functions remain largely unexplored in the basal angiosperm *Annona atemoya*, an economically significant species. This study aimed to elucidate the role of the WOX gene family in growth and regeneration in *atemoya* using genome-wide identification and functional characterization.

**Results:**

In the *atemoya* genome, 11 *WOX* genes were identified and phylogenetically classified into three distinct clades: Ancient, Intermediate, and Modern. This classification was confirmed by their conserved gene structures and motif compositions. A comparative genomic analysis revealed conserved synteny and purifying selection (Ka/Ks < 1) among *WOX* orthologs of *atemoya*, *Amborella*, and *Arabidopsis*, highlighting evolutionary constraints and essential functional roles. Analysis of promoters in *cis*-elements showed the enrichment of development- and hormone-responsive motifs, indicating that *AaWOX* genes are integrated in conserved regulatory networks. Tissue-specific expression profiling revealed divergent spatiotemporal patterns. *AaWOX1* and *AaWOX3* were highly expressed in shoot tissues and during shoot regeneration. *AaWOX11/12* and *AaWOX5/7* were preferentially expressed in roots, but only *AaWOX11/12* was significantly upregulated during root regeneration. Notably, *AaWUS* was almost undetectable in normal roots but became strongly induced upon callus formation, implying a role in de-differentiation and cellular reprogramming.

**Conclusions:**

Our study provides a comprehensive genome-wide analysis of the *AaWOX* family in *atemoya*, revealing its clade-specific evolutionary conservation and putative functional divergence during organ development and regeneration. These findings highlight the pivotal roles of *WOX* transcription factors in coordinating regenerative responses in a basal angiosperm and establish a critical foundation for the genetic improvement and biotechnology applications in *Annona atemoya*.

**Supplementary Information:**

The online version contains supplementary material available at 10.1186/s12870-026-08109-5.

## Introduction

Atemoya (*Annona atemoya*), a commercially valuable but understudied fruit tree, has complex growth and developmental traits that influence its breeding efficiency [1]. Although *WUSCHEL*-related homeobox (*WOX*) transcription factors are known to regulate meristem maintenance [2, 3], organogenesis [4, 5], and stress responses [6] in plant species, their roles in *atemoya* remain unexplored, representing a significant knowledge gap in this economically crop.

WOXs are plant-specific homeobox transcription factors. Their defining feature is a conserved 60-amino-acid DNA-binding homeodomain, and most members additionally possess a distinct C-terminal WUS-box motif, which is crucial for functional specificity [7, 8]. In *Arabidopsis thaliana*, the 15 family members are phylogenetically classified into three clades with distinct functional specializations [8, 9]. The Modern Clade (*WUS*, *WOX1-7*) is primarily responsible for stem cell maintenance and organ patterning in apical meristems. Within this clade, WUS is essential for stem cell identity in the shoot apical meristem (SAM) [10]; *WOX1* balances stem cell pluripotency and differentiation [11]; *WOX3* is involved in lateral organ development [12]; *WOX4* regulates vascular cambium activity and suppresses premature xylem differentiation [13, 14]; and WOX5 plays a key role in specifying root apical meristem (RAM) identity [4, 15]. The Intermediate Clade (*WOX8*, *WOX9*, *WOX11*, *WOX12*) plays crucial roles in early embryogenesis and stress-responsive regeneration. *WOX9* is vital for embryo development and maintaining cell division [16], and *WOX11/12* promote cell reprogramming during de novo root organogenesis [17] Finally, the Ancient Clade (*WOX10*, *WOX13*, *WOX14*) is associated with specialized developmental processes, including floral organ abscission and seed maturation [7]. This comprehensive functional diversification underscores the central and evolutionarily adapted roles of the WOX family in coordinating plant growth and development.

The absence of *WOX* genes in red algae (*Cyanidioschyzon merolae*) and their ubiquitous presence in land plants and green algae (*Ostreococcus lucimarinus* and *Ostreococcus tauri*) indicate that this gene family originated in the common ancestor of green plants [18, 19]. Based on the evolutionary progression of plants, the WOX gene family has undergone a clear trend of expansion in member numbers. Green algae, representing an early evolutionary stage, contain only a single WOX ortholog [19]. In basal angiosperms, the family size increases modestly, with *Amborella trichopoda*, wintersweet (*Chimonanthus praecox*), and *Liriodendron chinense* possessing 9, 13, and 11 members, respectively [20, 21]. This number varies significantly among core angiosperms. In monocots, rice (*Oryza sativa*) and maize (*Zea mays*) have 14 and 30 members, respectively [22]. Among eudicots, *Arabidopsis thaliana* contains 15 members [8], while soybean (*Glycine max*) exhibits a substantial expansion to 33 members [23]. A survey of Rosaceae species reveals a wide range from 6 to 40 members, with *Prunus domestica* (40), *Malus domestica* (18), and *Fragaria vesca* (15) at the higher end, and *Rubus occidentalis* (6) at the lower end [24]. This pattern suggests that lineage-specific whole-genome duplications and subsequent gene retention or loss events have played a major role in shaping the diverse complement of WOX genes across plant species. Despite these advances, there are many gaps in our understanding of the evolutionary trajectory of the WOX family. It remains unclear when and how functional divergence arose among *WOX* members, particularly whether subfunctionalization or neofunctionalization occurred early (e.g., prior to monocot–dicot divergence) or later during lineage-specific adaptations. Resolving this timeline is essential for elucidating the interplay between *WOX* gene diversification and plant developmental evolution.

Recent genomic advancements now make such investigations feasible. At present, genome assemblies of at least five Annona species have been reported or are publicly accessible, including published genomes for *A. muricata*(2021) [25], *A. glabara*(2022) [26], *A. montana*(2023) [27], *A. cherimola*(2023, 2024) [28, 29], and *A. squamosal*(2024) [30–32]. To further enable comparative and functional studies, we have generated a high-quality genome assembly for *A. atemoya* (unpublished data). Leveraging these genomic resources, a systematic investigation of the WOX family in this ancient fruit tree will not only reveal the ancestral regulatory mechanisms of plant development but also provide novel insights into the adaptation of WOX-mediated transcriptional networks during angiosperm evolution.

## Results

### Identification of *WOX* sequences in *Annona Atemoya*

We used the full-length sequences of 15 and 9 *WOX* family members in *Arabidopsis thaliana* and *Amborella trichopoda*, respectively, from TFTB (https://planttfdb.gao-lab.org/index.php) as BLAST queries to obtain the sequences of WOX family members in *Annona atemoya*. Subsequently, redundant sequences were removed based on their identification numbers (Pfam: PF00046) and chromosome locations, resulting in 11 *WOX* genes (Table 1). These genes encoded proteins with 175–602 aa, an isoelectric point (pI) range of 5.62–9.64, and the molecular weights of 20.02–69.99 kDa. Subcellular localization prediction showed that all *AaWOX* proteins localized in the nucleus (Table 1).

**Table 1:** *Annona atemoya* WOX gene identified in this study

Gene name	Gene ID	Chromosome	ORF(bp)	Length(aa)	pI	Mw(kDa)	Aliphatic index	Hydropathicity index	Subcellular localization
AaWUS	AsquamosaA03T001665.1	Chr3	759	263	7.17	29.29	47.49	-0.786	Nucleus
AaWOX1	AsquamosaA03T000590.1	Chr3	948	326	9.04	36.57	59.69	-0.688	Nucleus
AaWOX2	AsquamosaA02T003833.1	Chr2	747	259	5.63	28.86	64.86	-0.554	Nucleus
AaWOX3	AsquamosaA03T002294.1	Chr3	588	206	9.4	23.66	50.73	-0.884	Nucleus
AaWOX4	AsquamosaA06T002169.1	Chr6	711	247	8.86	27.52	57.29	-0.879	Nucleus
AaWOX5/7	AsquamosaA01T001532.1	Chr1	495	175	9.28	20.02	57.94	-0.782	Nucleus
AaWOX9	AsquamosaA02T002529.1	Chr2	1065	365	6.33	40.37	59.89	-0.588	Nucleus
AaWOX13	AsquamosaA03T002847.1	Chr3	900	310	5.57	35.24	62.9	-0.872	Nucleus
AaWOX11/12	AsquamosaA03T001199.1	Chr3	801	277	5.62	30.53	66.57	-0.261	Nucleus
AaWOX11/12-like 1	AsquamosaA01T004600.1	Chr1	1122	384	9.64	42.24	70.6	-0.364	Nucleus
AaWOX11/12-like 2	AsquamosaA01T004599.1	Chr1	1776	602	7.15	69.99	65.28	-0.725	Nucleus

### Phylogenetic and synteny analysis of *WOX* genes in *Annona Atemoya* and other species

**Fig. 1:**
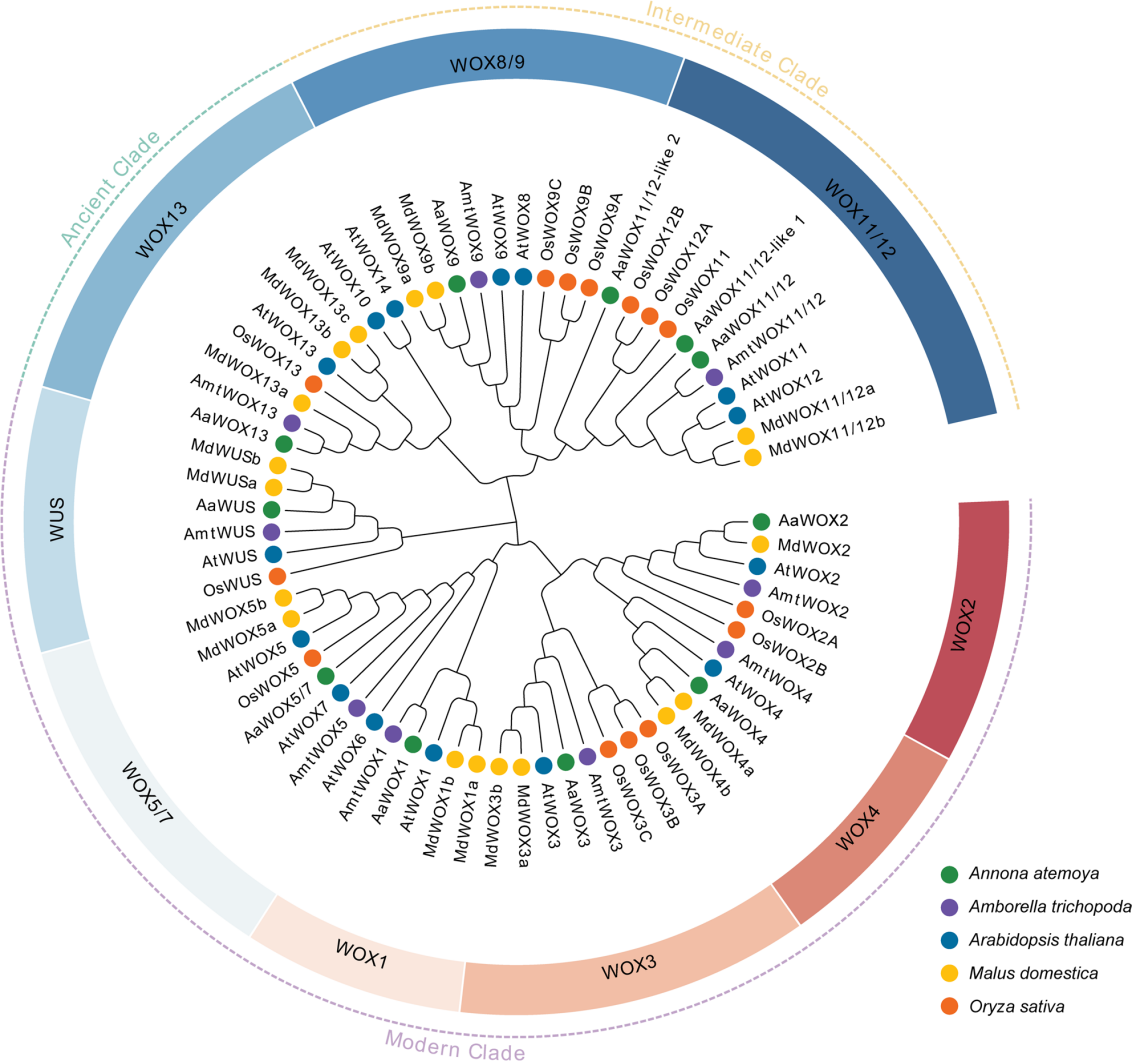
Phylogenetic analysis of *Annona atemoya*, *Malus domestica*, *Amborella trichopoda*, *Oryza sativa*, and *Arabidopsis thaliana* WOX proteins. *Annona atemoya*, *Malus domestica*, *Amborella trichopoda*, *Oryza sativa*, and *Arabidopsis thaliana* WOX proteins are indicated by green, yellow, purple, orange, and blue circles, respectively

To explore the evolutionary divergence of *WOX* genes, 67 WOX protein sequences from *Arabidopsis* (15), apple (*Malus domestica*) (18), *Amborella trichopoda* (9), *Oryza sativa* (14), and *atemoya* (11) were used to construct a phylogenetic tree (Fig. 1). *Atemoya* members were renamed based on the original Arabidopsis names clustered in the same clade. *WOX* members in each species were divided into three clades: Ancient, Intermediate, and Modern Clades. The Modern Clade was the largest in the tree and remained the largest in *atemoya* and the other species. The Ancient Clade only contained eight *WOX* members and was the smallest clade in each tested species (Fig. 1), indicating that WOX members were conserved during evolution.

**Fig. 2:**
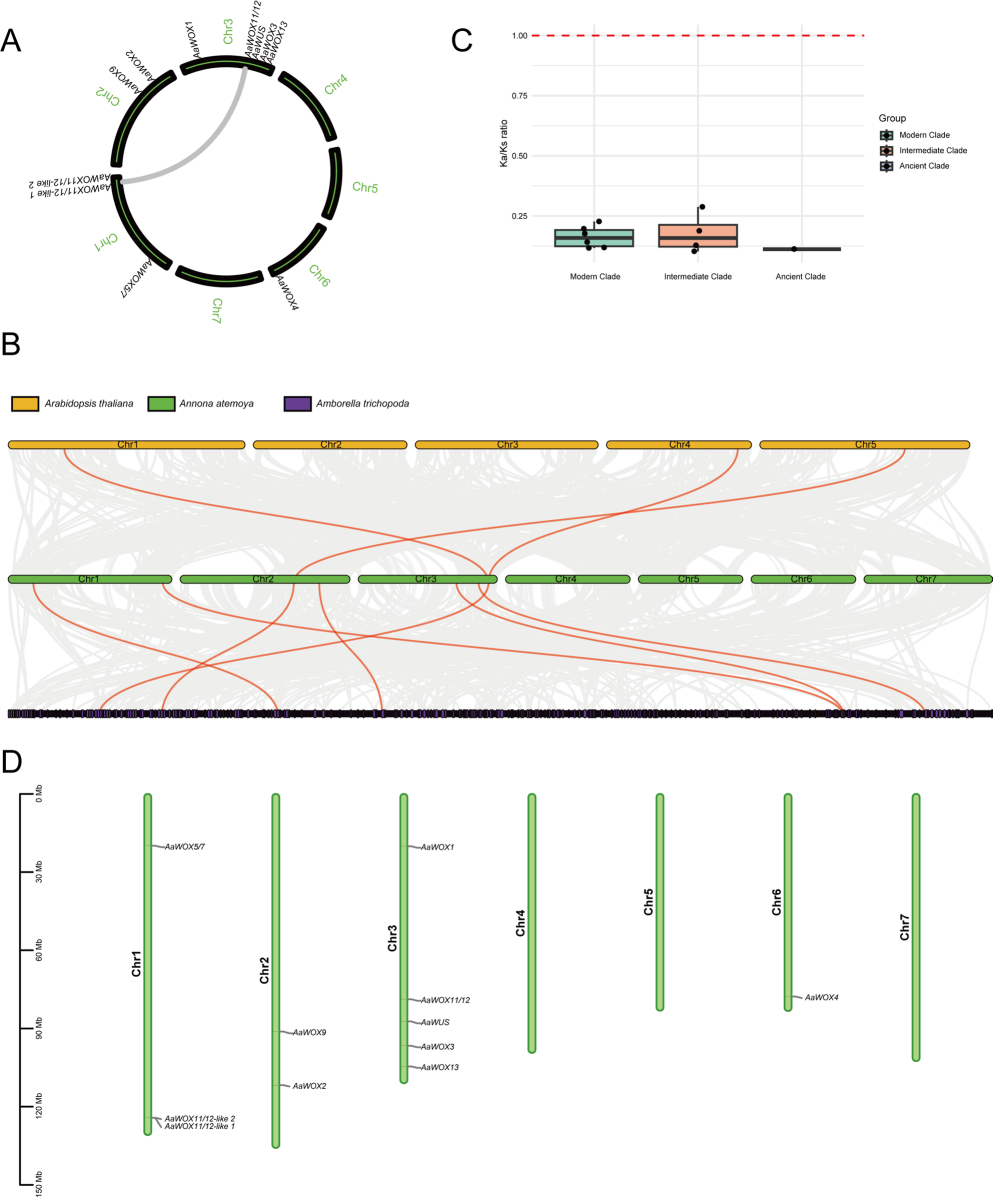
Chromosomal localization and synteny analysis of *AaWOX* family genes. **A**: Chromosomal location and synteny analysis of *AaWOX* genes. **B**: Synteny analysis of *Annona atemoya* with *Arabidopsis thaliana* and *Amborella trichopoda*. **C**: Ka/Ks value of *AaWOX* genes in different clades. **D**: Chromosome distribution of WOX gene family members in atemoya. The left axis displays the length of each chromosome, estimated in megabases (Mb)

Gene duplication, including whole genome, tandem, segmental, and gene duplications, occurs universally during plant evolution, producing homologous genes that share sequence similarities. Synteny analysis was performed to reveal the evolutionary characteristics of the *WOX* gene family. Intraspecies collinearity analysis revealed one pair of fragment duplication events in the members of the *AaWOX* gene family: *AaWOX11/12* and *AaWOX11/12 like 1* (Fig. 2A). *Arabidopsis thaliana* and *Amborella trichopoda* were selected for interspecies collinearity analysis with *Annona atemoya*. In the comparison between atemoya and *Arabidopsis*, three gene pairs exhibited collinear relationships (Fig. 2B). Similarly, seven pairs of genes had collinear relationships in the comparison between atemoya and *Amborella*, indicating a high degree of homology between the *AaWOX* genes of *atemoya* and the *AmtWOX* genes of *Amborella*, as both are primitive angiosperms (Fig. 2B), suggesting a close relationship between magnoliids and basal angiosperms.

To further analyze the evolution of the WOX gene family, Ka/Ks analysis was performed by comparing the nucleic acid sequences of genes in *Amborella* and *atemoya*. The Ka/Ks ratio was calculated for collinear and duplicated gene pairs between *Amborella* and atemoya, all of which had a Ka/Ks ratio of < 1 (0.19–0.60) (Fig. 2C), suggesting that purifying selective pressure occurred during WOX gene family evolution and that a conserved function is shared by these genes.

Based on genetic mapping, the 11 *WOX* gene family members in *atemoya* were located on four chromosomes (Fig. 2D). No *WOX* gene family members were identified on chromosomes 4, 5, and 7, and there was only one member on chromosome 6. Chromosomes 2, 1, and 3 had two, three, and five members of the *WOX* gene family, respectively. *WOX 11/12-like 1* and *WOX 11/12-like 2* were tandem duplicated on chromosome 1.

### Gene feature analysis of the AaWOX family

**Fig. 3:**
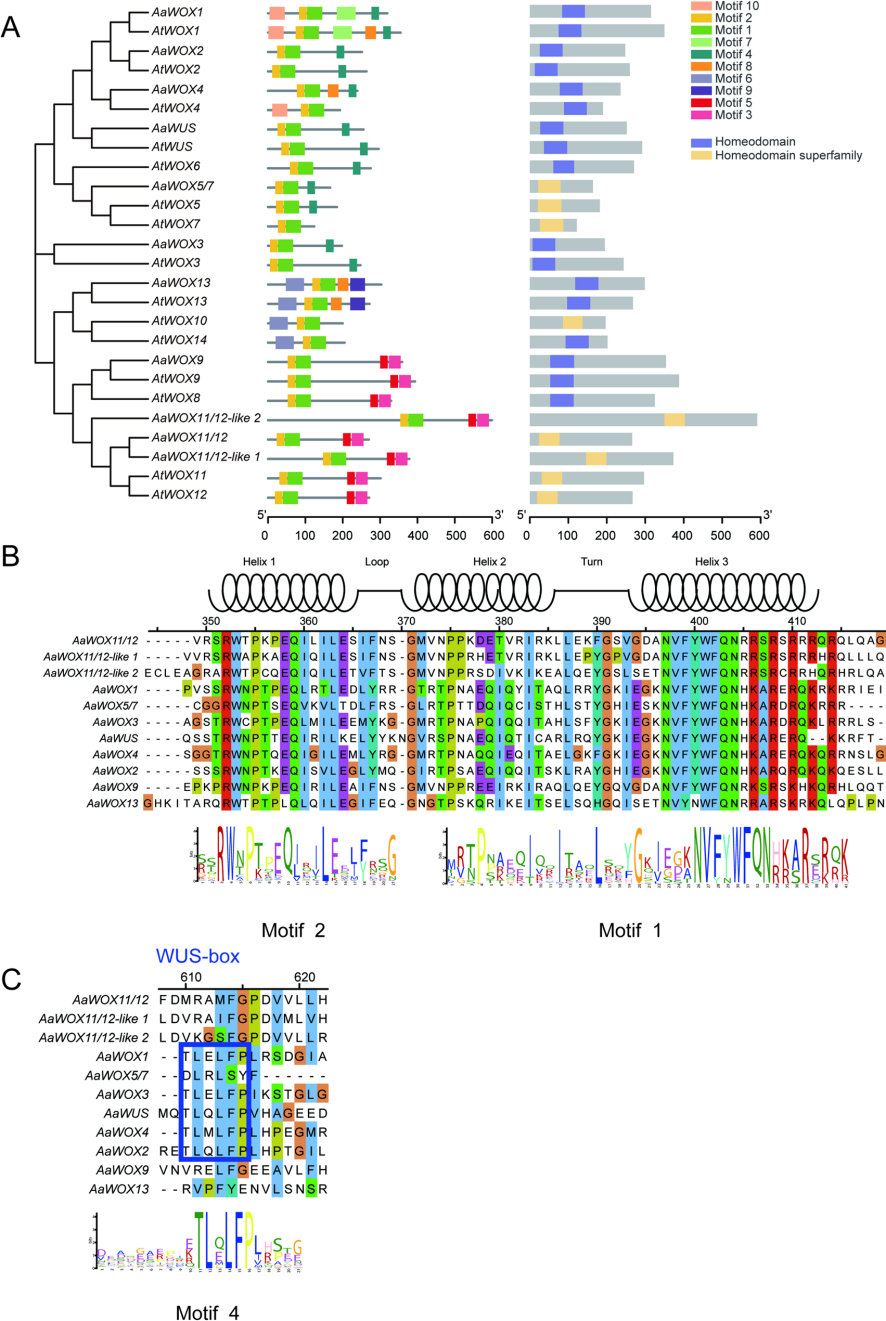
Sequence feature analysis of AaWOXs in *atemoya*. **A**: Analysis of conserved AaWOX motifs. Sequence size (bp) is indicated by the scale at the bottom of the figure. The “homeodomain” (pfam00046) is the sole member of the “homeodomain superfamily” (cl47488). **B**: Multiple sequence alignment of AaWOX protein homeodomains. **C**: Multiple sequence alignment of the WUS-box in AaWOX proteins

*WOX* members are plant-specific proteins containing a conserved homeodomain [33, 34]. To determine whether the domain was conserved among *AaWOX* members, these protein sequences were aligned to generate sequence logos. The alignment results showed that all *AaWOX* members contained a conserved homeodomain carrying a helix-loop-helix-turn-helix structure (Fig. 3A). Consistent with previous studies [35–37], representative motifs YNWFQNR, FYWFQNR, and FYWFQNH exist in homeodomains from the Ancient, Intermediate, and Modern Clades, respectively (Fig. 3B). Except *AaWOX5/7*, members in the Modern Clade also contain a WUS-box (Fig. 3C). *AaWOX* members in the Ancient and Intermediate Clades had no WUS-boxes, indicating that *AaWOX* members in different clades are involved in different biological processes in *atemoya*.

**Fig. 4:**
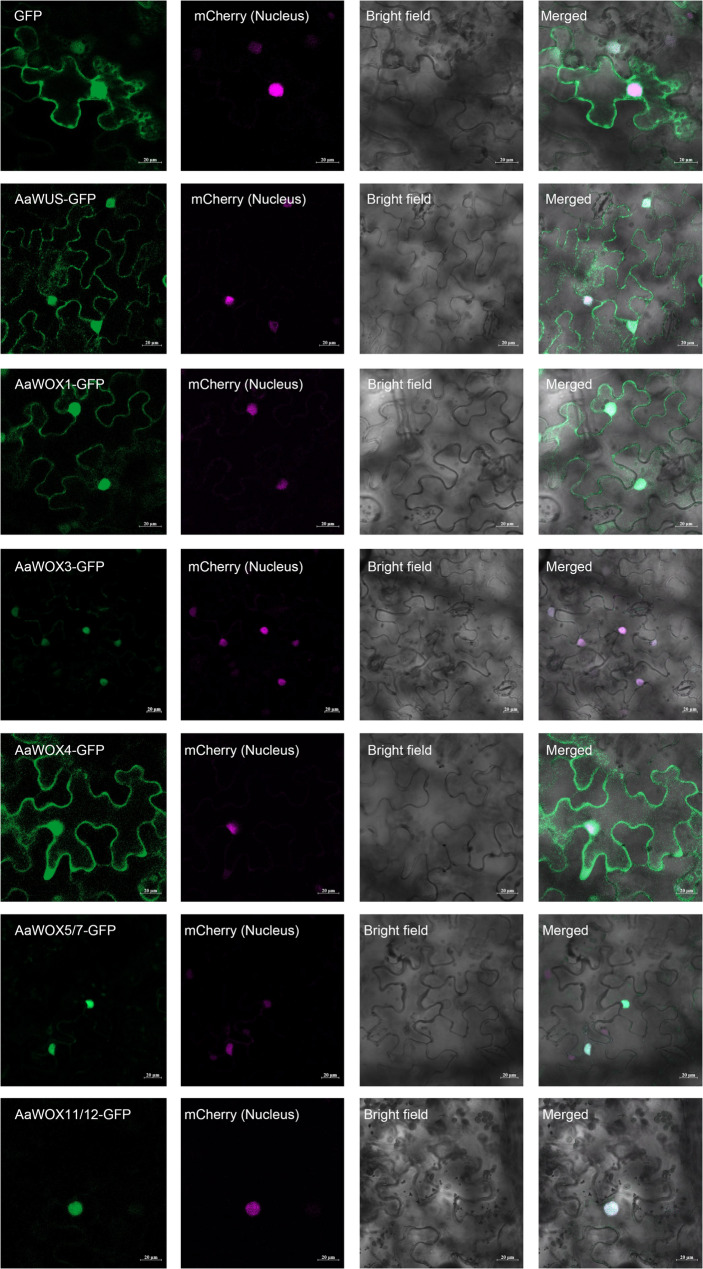
Subcellular localization of AaWOXs in tobacco leaves. GFP was fused to the C terminus of AaWOXs to yield AaWOXs-GFP fusion proteins. An mCherry-labelled nuclear marker (35 S: H2B-mCherry) was co-infiltrated with AaWOXs-GFP as a positive control, which is labeled with red florescence. The fluorescence signals in *Nicotiana benthamiana* leaves were detected by confocal microscopy 72 h after infiltration

Subcellular localization analysis revealed distinct patterns for the AaWOX-GFP fusion proteins. In the positive controls, the 35 S: H2B-mCherry marker was confined to the nucleus. AaWOX3-GFP, AaWOX5/7-GFP, and AaWOX11/12-GFP fusions similarly exhibited exclusively nuclear localization. In contrast, AaWUS-GFP, AaWOX1-GFP, and AaWOX4-GFP were detected in both the nucleus and cytoplasm (Fig. 4). These experimental observations contradict in silico predictions, which indicated exclusive nuclear localization for all AaWOX proteins (Table 1).

### *AaWOXs* promoter regions contain key *cis*-elements related to phytohormones and stress responses

**Fig. 5:**
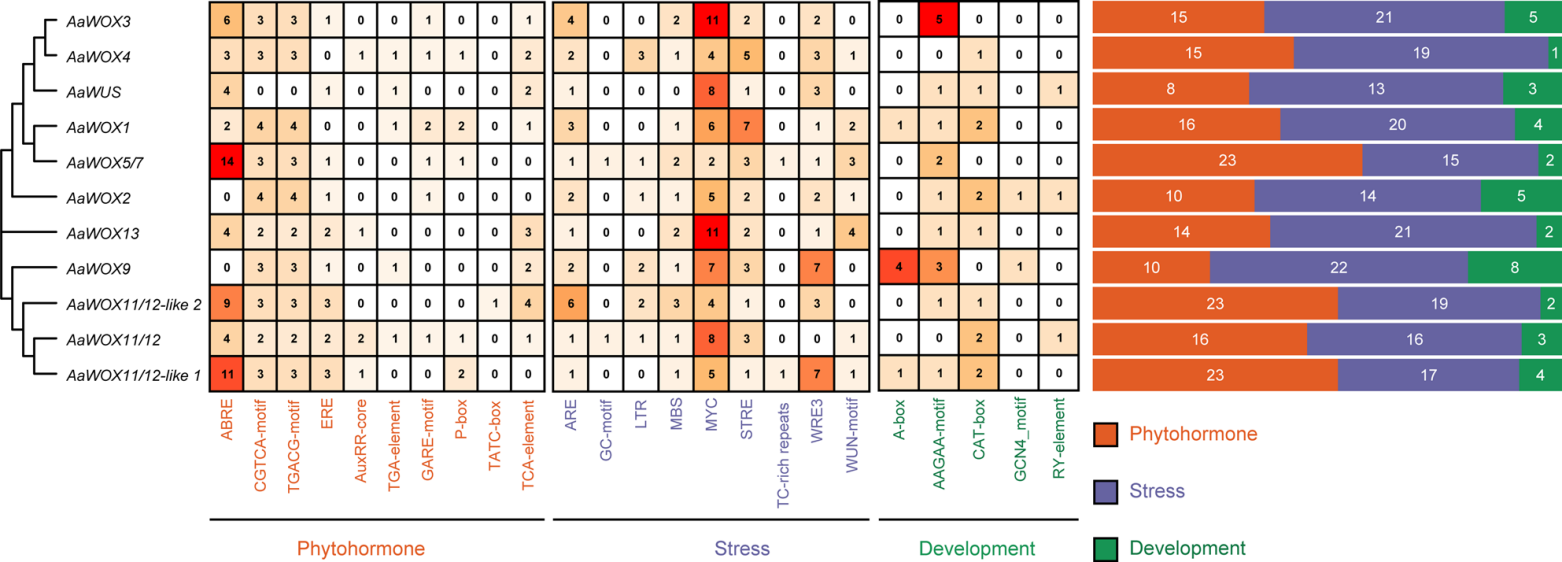
Analysis of promoter *cis*-acting elements in *AaWOXs*. Promoter regions 2,000 bp upstream of the *AaWOX* genes translation start sites were analyzed by PlantCARE. Boxed numbers denote the count of *cis*-elements within AaWOX promoters. Rectangles with different colors represented different *cis*-acting elements. Orange, purple, and green orthogons indicated phytohormone, stress, and development-related elements

*Cis*-elements are non-coding DNA sequences in gene promoter regions that are crucial for gene expression and widely involved in the regulation of plant growth, development, and stress responses. To better understand the regulatory network of *AaWOX* genes, their promoter regions were analyzed, revealing that they all contained *cis*-elements associated with plant growth and development, phytohormones, and stress responses. The response elements identified in the 2000-bp promoter region upstream of *atemoya WOX* family genes were classified into three categories based on their functions. Of these, 39, 173, and 197 elements were related to plant growth and development, phytohormone response pathways, and abiotic and biotic stress, respectively (Fig. 5). The most abundant *cis*-elements in the promoter regions of *AaWOX* family genes were salt stress response elements (MYC), abscisic acid response elements (ABRE), and growth hormone response elements (AAGAA-motif), suggesting that the *AaWOX* gene family is involved in a variety of pathways related to growth, development, and stress responses in *atemoya*.

### *AaWOX* expression patterns in *Atemoya*

**Fig. 6:**
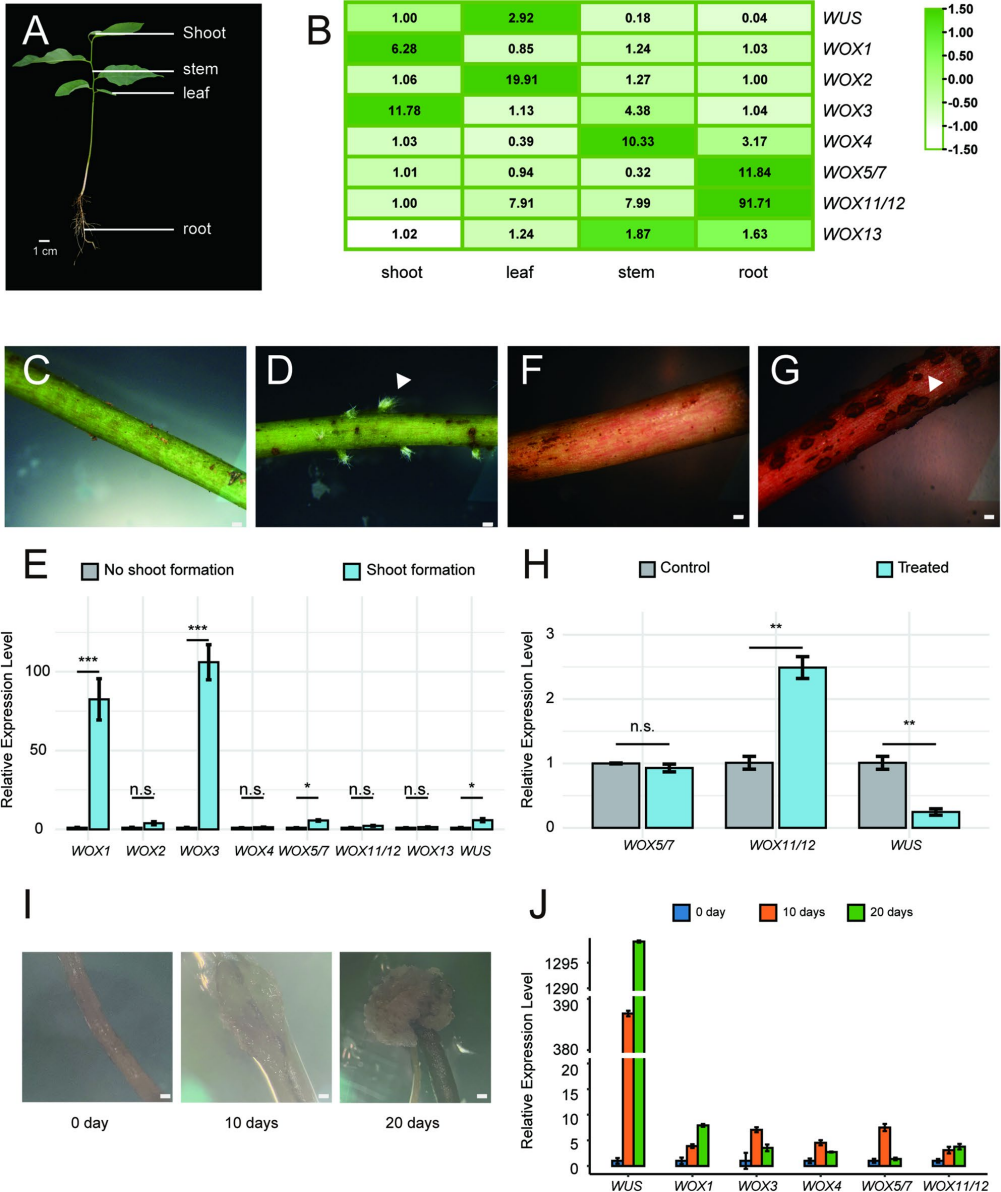
Expression profile analysis of *AaWOXs*. **A**: The shoot, stem leaf and root of atemoya seedling. Scale bars: 1 cm. **B**: Heatmap of the tissue-specific expression characteristics of *AaWOXs* in atemoya seedling. Samples were from shoot, leaf, stem, and root. Expression level of each AaWOX gene can be estimated based on scale on right. Dark green, light green and white indicate high, medium and low levels of gene expression, respectively. **C**: Atemoya seedling cut tips exhibited no shoot regeneration. Scale bars: 200 μm. **D**: Truncated atemoya seedling tips displayed prolific shoot regeneration. Scale bars: 200 μm. White triangles indicate the sites of shoot formation on the hypocotyl. **E**: Expression characteristics of *AaWOXs* during de novo shoot formation versus non-formation in decapitated atemoya seedling. Bars represent mean ± SD of three biological replicates; ns, *p* > 0.05; *p < 0.05; **p < 0.01; ***p < 0.001 (the unpaired Welch’s t-test). **F**: Absence of root formation from the hypocotyl after root excision, and one month of cultivation. Scale bars: 200 μm. **G**: De novo root emergence from the hypocotyl after root excision, inoculation with *Agrobacterium rhizogenes* strain R599, and one month of cultivation. Scale bars: 200 μm. White triangles indicate the sites of root formation on the hypocotyl. **H**: Expression characteristics of AaWOXs during de novo root formation versus non-formation in atemoya seedlings after root excision. Bars represent mean ± SD of three biological replicates; ns, *p* > 0.05; *p < 0.05; **p < 0.01; ***p < 0.001 (the unpaired Welch’s t-test). **I**: Different stages of callus induction by roots of atemoya. Scale bars: 200 μm. **J**: Expression characteristics of *AaWOXs* in callus at different stages. Bars represent mean ± SD of three biological replicates

To investigate the tissue-specific expression of *AaWOX* genes in atemoya, the expression patterns of all 11 *AaWOX* genes were evaluated using quantitative real-time polymerase chain reaction (qRT-PCR) in four tissues/organs, i.e., shoot, leaf, stem, and roots (Fig. 6A), with expression differences greater than 2-fold or less than 0.5-fold considered biologically significant. *AaWOX1* and *AaWOX3* were mainly expressed in shoots. *AaWUS* was relatively highly expressed in shoots and leaves. *AaWOX2* was specifically expressed in leaves, whereas *AaWOX4* was highly expressed in stems. *AaWOX5/7* and *WOX11/12* showed significantly high levels of expression in roots, and *AaWOX13* showed no obvious organ-specific expression patterns (Fig. 6B). *AaWOX9*, *AaWOX11/12-like 1*, and *AaWOX11/12-like 2* showed low expression in atemoya tissue. These results suggest that different *AaWOX* members exhibit distinct tissue-specific expression patterns, which may be correlated with their regulatory functions.

To further investigate the effect of the *AaWOX* member on tissue development in *atemoya* seedlings, the apical buds of atemoya seedlings were removed, and expression differences were detected between differentiated and undifferentiated shoot tissues. Compared with undifferentiated shoot tissues, the expression levels of *WOX1* and *WOX3* genes were significantly increased in differentiated shoot tissues (Fig. 6C–E).

*Atemoya* seedlings usually cannot regenerate root tissue after it is cut off, but after treatment with *Agrobacterium tumefaciens*, the seedlings formed new root tissue within 1 month from hypocotyl. To investigate the role of *AaWOX* members in the root regeneration process of *atemoya* seedlings, we profiled the entire AaWOX family and found that the differential expression of *AaWOX* members was determined between hypocotyl treated with *Agrobacterium tumefaciens* for 1 month and a control group. The *AaWOX11/12* gene was expressed in the treatment group, but the expression level of *AaWUS* decreased. There were no significant differences in the expression levels of *AaWOX5* (Fig. 6F–H).

*Atemoya* root tissue grew a visible callus after 10 days in callus induction medium. To determine the role of *AaWOX* members in atemoya callus formation, the expression levels of *AaWOX* genes were analyzed in callus tissues at different stages of induction. The *AaWUS* gene was significantly expressed at a high level (Fig. 6I, J).

## Discussion

### Lineage-specific functional diversification of WOX genes under evolutionary constraints in *Annona Atemoya*

The *WOX* gene family is a highly conserved transcription factor family in plants. The functions of the members of this family are essential for normal plant development [38–40]. However, only a few members have been characterized in plant species. Studies on the functions of *AaWOX* genes have been very limited, and the members of the *AaWOX* gene family have not been systematically identified or defined. In this study, we identified 11 *AaWOX* genes from the *atemoya* reference genome, and they were distributed on 7 *atemoya* chromosomes. We also performed bioinformatics analyses of the gene family, determining the gene structure, conserved motifs, *cis*-regulatory elements, and physicochemical properties of proteins, and predicted subcellular localization, providing a framework for further studies on this gene family.

In this study, *AaWOX* members were classified into three clades: Ancient, Intermediate, and Modern Clades (Fig. 1). As shown in previous studies [8], each AaWOX protein contained a homeodomain, and all members in the Modern Clade had a WUS-box domain, except AaWOX5/7 (Fig. 3), indicating that the homeodomain is highly conserved in WOX members.

Gene structure and motif analyses showed that the *WOX* gene family in *atemoya* had a unique motif, and all genes contained motifs 1 and 2 (homeodomain). Different branches also had their own unique themes. For example, motifs 3 and 5 were only present in the Intermediate Clade subfamily, and motif 4 was only present in the Modern Clade. Similarly, in *Arabidopsis thaliana*, the Ancient Clade had motifs 6 and 9, which were not present in the other evolutionary branches (Fig. 3A). This indicates that WOX genes in different evolutionary branches have different functions.

### Link between subcellular localization and functional diversification of *AaWOX* genes

We characterized the subcellular localization of AaWOXs to determine their functional divergence. Although AaWOX3, AaWOX5/7, and AaWOX11/12 exhibited strict nuclear confinement, supporting their canonical roles as transcription factors, AaWUS, AaWOX1, and AaWOX4 displayed dual nucleo-cytoplasmic partitioning (Fig. 4). This divergence suggests unconventional regulatory layers that may involve non-transcriptional functions or dynamic nucleo-cytoplasmic trafficking.

The cytoplasmic presence of AaWUS contrasts with the exclusively nuclear localization of WUS in wintersweet [20], alfalfa [6], rice [41], wheat [42], and tobacco [43]. This discrepancy may reflect species-specific regulatory mechanisms. We hypothesize that the cytoplasmic pool of AaWUS is linked to its intercellular mobility, with WUS undergoing synthesis-triggered translocation, as proposed by Yadav et al. (2011) [44]. Its transport signals and functional states within the cytoplasm require further investigation.

A comparative analysis revealed inconsistent subcellular patterning among WOX orthologs. Although AtWOX1 [11] and OsWOX3 [45] also localized to the cytoplasm, GmWOX1A [46], AtWOX3 [47], MsWOX3 [6], and MdWOX4/5/11 [48] remained strictly nuclear. Such variation implies functional diversification or differential post-translational regulation across species. While AaWOX5/7 is nuclear, studies of AtWOX5 have revealed that nuclear homogeneity can be redirected to nuclear bodies via protein interactions (e.g., with PLT3) [49], suggesting that nuclear WOX proteins can exhibit conditional compartmentalization. The cytoplasmic localization of some AaWOXs could indicate functional pools poised for regulation, degradation, or non-cell-autonomous signaling—features that challenge the nuclear paradigm of WOX function, which merits rigorous dissection.

### Lineage-specific expression divergence underpins functional specialization of *AaWOX* genes in *Annona Atemoya*

Unlike animals, many plants can regenerate and form an entire plant body from various tissues or organs or even from a single somatic cell [50]. Among plant regeneration types, de novo organogenesis, in which adventitious roots and shoots are formed from wounded or detached plant tissues or organs, is frequently used in research and biotechnological breeding, as it is a simple and robust in vitro plant culture method [51]. Expression pattern analysis showed that a few of these *AaWOX* genes were expressed in specific organs, indicating that these genes are may be involved in different developmental processes.

Previous studies have shown that *AtWOX1* is essential for SAM maintenance, with its overexpression causing severe meristematic defects [11]. The role of *WOX3/PRS* has been predominantly characterized in angiosperms [47], where it is instrumental in floral organ and leaf development. In Norway spruce, this function is not an angiosperm novelty but rather a fundamental, ancestral trait that predates the divergence of gymnosperms and angiosperms [52]. Our expression analysis revealed significantly elevated *AaWOX1* and *AaWOX3* transcript levels in regenerative buds (Fig. 6E), suggesting their putative roles in regulating shoot development. However, further investigation is needed to determine whether these paralogs have functionally diverged in the somatic organogenesis of *atemoya*.

*AaWOX5/7* and *AaWOX11/12* have high expression levels in root tissues, but only *AaWOX11/12* has high expression in induced root tissues. Previous studies have shown that *OsWOX11* is expressed in emerging crown roots and later in cell division regions of the RAM in rice [53], and *AtWOX11* and *AtWOX12* are involved in de novo root organogenesis in Arabidopsis [17]. These results are consistent with the expression of the *WOX11/12* gene in the present study (Fig. 6H), indicating that *AaWOX11/12* may play an important role in the root regeneration process of atemoya seedlings. In the phylogenetic tree, *AaWOX5/7* belongs on the same evolutionary branch as *AtWOX5* and *AtWOX7* (Fig. 1). *AtWOX5* maintains stem cells in roots [4]. *AtWOX7* inhibits lateral root formation [54]. *MdWOX5* expression significantly enhances the transformation efficiency and stability of apple [55]. High *AaWOX5/7* expression in roots may be related to the maintenance of RAM, rather than root tissue regeneration.

The canonical role of *WUSCHEL* (*WUS*) as a key regulator for maintaining the stem cell population in the SAM is well established in eudicot models, such as *Arabidopsis thaliana* [10] and *Solanum lycopersicum* (tomato) [56]. Our data from *Annona atemoya*, a basal angiosperm with high *WUS* expression in undifferentiated callus, strongly support the hypothesis that the ancestral function of *WUS* was the promotion and maintenance of a pluripotent, undifferentiated cell state. However, the functional divergence of *WUS* in rice (*Oryza sativa*), a monocot, in which it has been reported to be dispensable for SAM maintenance, highlights the significant evolutionary reprogramming of this conserved genetic module within the monocot lineage [57]. We propose an evolutionary trajectory for the WUS gene function. The ancestral WUS, as retained in *Annona*, primarily functioned as a broad-spectrum promoter of cell pluripotency, as shown by its activity in callus cells. In core eudicots, such as Arabidopsis and tomato, this function became specifically refined and canalized to maintain a highly organized stem cell niche within the SAM. In contrast, this role in the SAM in the monocot lineage was co-opted by other genetic factors, freeing WUS to evolve novel functions, perhaps in response to the distinct developmental architecture of monocots.

A striking observation from *cis*-element analysis is the pervasive enrichment of MYC-binding motifs in the promoters of all *A. atemoya* WOX genes (Fig. 5). Because MYC proteins are core executors of jasmonate signaling [58], we speculate that the JA pathway may contribute to the transcriptional modulation of the WOX family. These observations place the WOX family at the putative nexus of environmental sensing and developmental output, where it may function as a regulatory hub that converts stress cues into adaptive growth responses. Elucidating the underlying molecular circuitry warrants targeted investigation.

## Conclusion

In this study, we systematically identified and characterized 11 *AaWOX* gene family members in the basal angiosperm *Annona atemoya*. Our comprehensive analysis revealed that these *AaWOX* genes are highly conserved and underwent strong purifying selection during evolution. All identified proteins contained the characteristic homeodomain, confirming their functional identity as members of the WOX gene family. Subcellular localization predictions showed that AaWOX proteins likely function in the nucleus or in both the nucleus and cytoplasm, consistent with their putative roles in transcriptional regulation. Expression profiling demonstrated the distinct spatial and temporal expression patterns of AaWOX genes among tissues, including buds, roots, and callus, suggesting their potential specialized roles in key developmental processes, such as organogenesis and the maintenance of pluripotent cell states. These findings provide valuable insights into the evolutionary conservation and functional diversification of the WOX family in basal angiosperms and establish a crucial foundation for future functional studies aiming to manipulate regeneration and development in Annona and related species.

## Materials and methods

### Plant materials

*Annona atemoya* Hort. Cv. African Pride (AP) was planted in the National Field Genebank for Tropical Fruit at the South Subtropical Crops Research Institute, Chinese Academy of Tropical Agricultural Sciences, Zhanjiang, Guangdong, China. The orchard is located at 21°12′N,110°4′E. The *atemoya* seedlings were grown in a standard glass greenhouse. Irrigation and pest control were performed following standard procedures.

### Gene identification

The full-length sequences of 15 *AtWOX* and 9 *AmtWOX* members obtained from PlantTFTB 4.0 [59] (https://planttfdb.gao-lab.org/index.php) were used as queries for BLASTp searches to identify *AaWOX* genes in the *atemoya* genome. The sequences of the candidate members were then analyzed using SMART [60] (http://smart.embl-heidelberg.de) and Pfam [61] (http://pfam.xfam.org) to exclude members that did not contain the complete homeodomain.

### Bioinformatics analysis of *AaWOX* family members

The amino acid sequences of *AaWOX* members in atemoya were obtained from the *Annona atemoya* genome, and the molecular weights and pIs of these members were determined using the ExPASy Proteomics Server [62] (https://web.expasy.org/protparam/). Gene structure analysis was performed using the online tools CDD [63] (https://www.ncbi.nlm.nih.gov/Structure/bwrpsb/bwrpsb.cgi) and MEME [64] (https://meme-suite.org/meme/tools/meme).

### Chromosomal localization and visualization of *WOX* genes

The chromosomal positions of *WOX* genes in the atemoya genome were extracted from the General Feature Format (GFF3) annotation file. The chromosome number, start position, and end position of each *AaWOX* gene were recorded. TBtools II (version 2.376) [65] was used to generate a linear chromosomal map, illustrating the distribution of *AaWOX* genes across the *atemoya* genome.

### Evolutionary tree analysis and synteny relationship analysis of *WOX* in different species

The full-length sequences of *AaWOX* family members were aligned using ClustalW in MEGA11(version 11.0.13) [66], and this alignment was used to generate the phylogenetic tree with the neighbor-joining method. Poisson correction, pairwise deletion, and a bootstrap with 1000 replicates were conducted. The Multiple Covariance Scanning Toolkit (MCScanX) integrated in TBtools II (version 2.376) [65] was used to analyze the collinearity and evolutionary relationships of WOX gene families across multiple species.

### Calculation of Ka/Ks values

Ka and Ks values represent the numbers of nonsynonymous substitutions per non-synonymous site and synonymous substitutions per synonymous site, respectively. The Ka and Ks values were calculated using the KaKs Calculator 2.0 [67] (https://ngdc.cncb.ac.cn/biocode/tools/BT000001, last accessed on May 25, 2022) with the GY-HKY model, and the ratios were calculated using DnaSP5 [68].

### Subcellular localization analysis of AaWOX proteins

*AaWUS*, *AaWOX1*, *AaWOX3*, *AaWOX4*, *AaWOX5* and *AaWOX11* coding sequences, without the stop codon, were obtained and inserted into the pGreenII GFP vector to express the corresponding AaWUS–GFP, AaWOX1–GFP, AaWOX3–GFP, AaWOX4–GFP, AaWOX5–GFP and AaWOX11–GFP fusion proteins. The nuclear marker 35 S: H2B-mCherry was labelled by mCherry and co-infiltrated with AaWOXs–GFP into tobacco (*N. benthamiana*) leaves [69]. The fluorescence signal was detected using a confocal microscope 72 h after the infiltration. The primers used are listed in Table S1.

### Identification of *cis*-acting elements in *AaWOX* promoters

The 2-kb region upstream of each AaWOX gene was obtained from the atemoya genome as the promoter region. The *cis*-acting elements were predicted on the Plant-CARE website [70] (http://bioinformatics.psb.ugent.be/webtools/plantcare/html/) and classified using Excel.

### Gene expression analysis

Total RNA was extracted using a Polymer-Rich Plant RNA Extraction Kit (Cinotohi, Changsha, China), and cDNA was synthesized using TransScript One-Step gDNA Removal and cDNA Synthesis SuperMix (AH311; TransGen Biotech, Beijing, China). The qRT-PCR was performed in a 10 µL reaction volume using Hieff UNICON^®^ Universal Blue qPCR SYBR Green Master Mix (11184ES03, YEASEN, Shanghai, China) on the ABI Quant Studio™ 6 Flex System (Applied Biosystems, Foster City, CA, USA). Each reaction contained: 5 µL of 2×Premix, 0.3 µL of each forward and reverse primer (10µM), 0.5 µL of cDNA template (diluted 10-fold), and 3.9 µL of nuclease-free water. Primers were designed with Primer Premier 5.0. The atemoya *AaActin* (*AsquamosaB01T001612.1*) gene was used as the reference gene. There were carried three biological replicates under the following conditions: 95℃, 10 min; 40 cycles (95℃, 15 s; 55℃, 60 s). All primer sequences used in this study are listed in Table S1. The statistical analysis was performed using R (version 4.3.1), including the assessment of the statistical significance of the expression differences in various organs of each analyzed tomato species using the unpaired t-test with Welch’s correction. Each experiment included three technical replicates and at least three biological replicates.

For gene expression pattern analysis, the *atemoya* roots, stems, leaves, and shoots were harvested for RNA isolation. Two-month-old *atemoya* seedlings were decapitated (tip removal) and cultured for 2 months to induce adventitious shoots. To induce adventitious roots, 2-month-old atemoya seedlings were root-excised, infected with *Agrobacterium rhizogenes* strain K599 (OD_600_ = 0.8) in infiltration buffer (0.05 M MES, 2 mM Na_3_PO_4_, 0.5% (w/v) D-glucose, and 0.1 mM acetosyringone) for 30 min under standard vacuum conditions [71], and cultured for 1 month. For callus induction, 2-month-old *atemoya* seedlings were surface-sterilized (70% ethanol for 15 min, followed by 2% NaClO for 20 min), and their roots were aseptically excised. Root explants were cultured on callus induction medium (B5 medium supplemented with 2.0 mg/L 2,4-D and 1.0 mg/L 6-BA) at 25 °C in the dark [72]. Callus samples were collected 0, 10, and 20 days after treatment for further analysis.

## Supplementary Information

Supplementary Material 1.

## Data Availability

All data used during the current study are included in this published article or are available from the corresponding author on reasonable request.
